# Brillouin microscopy in cancer research: a review

**DOI:** 10.1117/1.JBO.30.12.124509

**Published:** 2025-10-16

**Authors:** Nathan Falkner, Meryem-Nur Duman, Zahra Zabolizadeh, Hadi Mahmodi, Chenjun Shi, Jitao Zhang, Thomas R. Cox, Irina Kabakova

**Affiliations:** a University of Technology Sydney, School of Mathematical and Physical Sciences, Ultimo, New South Wales, Australia; b Michigan State University, Institute for Quantitative Health Science & Engineering, Department of Biomedical Engineering, East Lansing, Michigan, United States; c Garvan Institute of Medical Research, Sydney, New South Wales, Australia; d UNSW Sydney, School of Clinical Medicine, Sydney, New South Wales, Australia

**Keywords:** Brillouin microscopy, Brillouin imaging, optical elastography, cancer detection

## Abstract

**Significance:**

Cancer is one of the leading diseases worldwide, continuing to pose a significant financial burden to national health systems and taking lives. These drive the development of early-stage cancer diagnostics, which is believed to be a crucial step in improving patients’ life expectancy and long-term outcomes of cancer treatments.

**Aim:**

In this review, we explore the potential of a label-free technique known as Brillouin microscopy, a type of optical elastography, emerging as a promising candidate for early-stage cancer screening.

**Approach:**

We discuss the main principles of this advanced imaging technology and provide a thorough analysis of all known Brillouin microscopy reports in application to cancer research and diagnostics. In our analysis, we focus on the mechanobiological aspect of the disease and draw conclusions based on four main sample types: cell cultures, cells in microfluidic environments, organoids, and excised tissues.

**Results:**

We review recent advancements in cancer detection, finding that the technique can consistently biomechanically delineate between healthy and unhealthy cells, and organoids and tissues across multiple cancer types. We also present strides made in imaging mechanical changes in cancer during varying stages of progression, treatment, and regression.

**Conclusions:**

We conclude this review with our perspective on the key developments required for technology’s translation into the clinical realm, including measurement standardization, inclusion of statistical and artificial intelligence methods into data analysis and automated diagnosis, and further hardware developments needed for *in situ* and *in vivo* micromechanical measurements.

## 1 Introduction

Cancer continues to pose a major health challenge worldwide, with the latest report from the World Health Organization indicating that nearly 20 million people were diagnosed with various types of cancer in 2020.^1^ The global 5-year prevalence exceeded 53 million cases, and cancer accounted for nearly 10 million deaths in the same year.^1^ Significant progress has been made in the early diagnosis of cancer, which is crucial for improving patients’ life expectancy and survival rates. Advances in artificial intelligence (AI)-assisted screening using MRI and CT imaging,^2^ label-free optical techniques (such as Raman spectroscopy, fluorescent probes, and two-photon imaging),^3^^–^^5^ liquid biopsy testing,^6^ biomarker detection (e.g., proteins, DNA, and RNA molecules, such as CA-125),^7^ and genomic profiling^8^ have all contributed to this progress. However, several major challenges persist, which can be broadly categorized as follows: (1) early detection of solid tumors remains difficult, particularly for pancreatic, ovarian, and brain cancers, which are often diagnosed late due to a lack of early symptoms or reliable screening tools; (2) the sensitivity and specificity of liquid biopsy and biomarker testing are still insufficient to reliably detect tumors, especially in their early stages; (3) the spatial and temporal heterogeneity of tumors complicates both diagnosis and treatment evaluation;^9^ and (4) the influence of the chemical, mechanical, and biomolecular aspects of the tumor microenvironment on cancer cell growth and migration is still not well understood, limiting the development of effective treatments and preventing tumor progression.

The last two categories—the tumor microenvironment and its inherent heterogeneity—are closely intertwined and should be considered together when investigating the relationship between a tumor’s biomechanical properties and the mechanosensitive responses of its cells. This relationship is fundamental to mechanobiology, which examines how physical forces and the mechanical properties of cells and tissues influence their biological functions. In fact, many diseases are found to alter the micromechanical state of tissues and cells, including respiratory conditions (lung fibrosis, emphysema, etc.),^10^ diseases of connective tissues (e.g., osteoarthritis^11^), and cancers,^12^^–^^14^ thus providing a pathway for micromechanical mapping techniques to be used to assist biomedical diagnostic tools.

In the context of cancer, mechanobiology takes on critical significance as the altered mechanical properties of the tumor microenvironment (TME) will directly influence cancer progression and therapeutic resistance. Changes in the stiffness of the extracellular matrix (ECM) surrounding cells found within the tumor, often driven by excessive matrix deposition and crosslinking, create a biomechanically distinct niche that can both promote and restrict disease progression.^15^ This biomechanical remodeling is typically transduced by integrins into cells (including cancer cells, stromal cells, and immune cells), where it alters downstream signaling processes through a series of effector proteins to modulate cell phenotype, including proliferation, survival, migration, invasion, and stemness. Importantly, the biomechanical heterogeneity observed within and between tumors—where stiffness can vary significantly between the invasive front, tumor core, and surrounding tissue—creates distinct microenvironments that contribute to diverse cellular phenotypes and treatment responses within a single tumor.

The bidirectional relationship between cells present within the tumor and ECM biomechanics presents both challenges and opportunities for cancer therapy. Cancer cells not only respond to biomechanical cues but also actively remodel their surroundings through the secretion of a wide repertoire of matrix molecules, as well as matrix-modifying enzymes. This creates feedback loops where initial changes in tissue biomechanics promote further biomechanical alterations and can reinforce and even accelerate changing phenotypes. Targeting the mechanosensitive pathways as well as modulating the biomechanical properties of the TME represents a promising therapeutic strategy. However, effective implementation requires precise, non-destructive methods to characterize mechanical properties at cellular and subcellular scales across tumor regions.

Understanding cell mechanosensing and mechanotransduction requires measurements of physical forces as well as the viscoelastic properties of intra- and extracellular environments. Several direct and indirect techniques have been developed to fulfil this goal, each with its advantages and limitations. Atomic Force Microscopy Nanoindentation remains the gold-standard technique of micro- and nano-mechanical characterization of cells, tissues, and biological materials.^16^ Although this technique can directly assess Young’s modulus of a monolayer of cells or a tissue sample, it is limited to surface measurements and thus does not produce reliable results for measurements of 3D organoids or bulk tissues. Optical coherence elastography (OCE) is another method, which combines the speed of optical coherence tomography with the convenience of elastography techniques.^17^^,^^18^ This method is currently growing in popularity, especially with the introduction of shear-wave optical coherence elastography^19^ to assess shear mechanical properties of tissues and cellular environments not accessible by other optical techniques. The limitation of OCE is in its spatial resolution, which so far exceeds 10 micrometers,^20^ meaning the technique is too coarse for quantifying mechanical properties at the single cell scale.

**Fig. 1:**
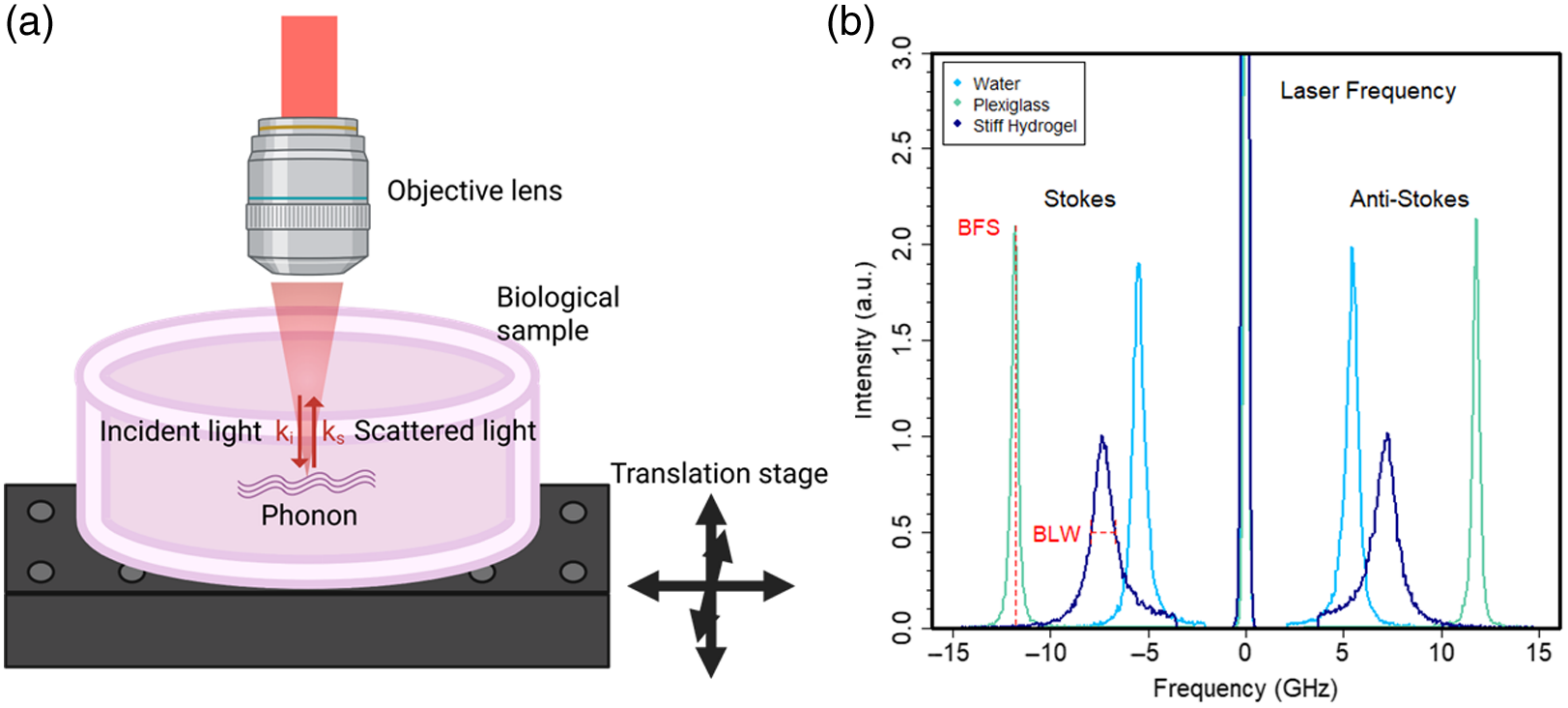
(a) Schematic of Brillouin light scattering within a biological sample made in Biorender. (b) Multiple spectra of varying common materials measured by a Brillouin Microscope, showing Brillouin Stokes and anti-Stokes peaks for several samples: water (light blue), stiff hydrogel (dark blue), and plexiglass (green).

Subcellular spatial resolution and 3D measurement capability can be provided by another optical elastography method, known as Brillouin microscopy.^21^ In this type of advanced microscopy, a focused beam of light interacts with biological material, producing an inelastically scattered signal [Fig. 1(a)]. The latter is detected using a specialized spectrometer or a heterodyne-type detection scheme, resulting in direct mapping of several physical parameters, Brillouin frequency shift, and linewidth. These parameters can be linked to the elasticity tensor and material viscosity (provided additional properties of the sample such as refractive index and density are known or can be reliably estimated) or be used as stand-alone characteristics of the material, enabling relative comparison between different cellular regions, control and treatment sample groups.^21^

In this review, we focus specifically on the emerging application of Brillouin microscopy in cancer research and diagnostics. We provide a detailed overview of published reports in this field of research together with critical assessment of the method’s benefits and limitations. We propose and address a central question: can the biomechanical fingerprint of cancer, as revealed through Brillouin microscopy, provide the missing link between basic research and clinical application? Technology’s ability to non-invasively probe the viscoelastic properties of living tissues offers a compelling new avenue to monitor disease progression and therapeutic response. The journey from benchtop Brillouin systems to clinical adoption presents significant challenges, but as we will demonstrate, recent technological breakthroughs are rapidly closing this gap. We conclude by giving our perspective on the areas of development in Brillouin microscopy which are required before the technique can become a standard diagnostic tool in cancer research and therapeutic applications, bringing us closer to a future where mechanical phenotyping becomes an integral component of precision oncology.

## 2 Brief Overview of Brillouin Microscopy

Brillouin light scattering (BLS) is a process in which light (photon) interacts with a high-frequency (GHz) pressure wave (phonon) within a medium, leading to the exchange of energy between a photon and a phonon and, consequently, producing the scattered light shifted in frequency by exactly the phonon frequency known as Brillouin frequency shift (BFS, $\Omega_{B}$). In most Brillouin microscopy experiments, the incident and scattered light are passing through the same objective lens as depicted in schematic Fig. 1(a), meaning the angle between the incident and the scattered beams is 180 deg. In this so-called back-scattering configuration, the BFS can be simply expressed as

$$\Omega_{B}=2n\lambda_{0}^{-1}\sqrt{\frac{M^{\prime }}{\rho }}.$$ (1)

Here $\lambda_{0}$ is the wavelength of light in free-space, n is the medium refractive index, ρ is its mass density and $M^{\prime }$ is the longitudinal storage modulus. Equation (1) suggests that $\Omega_{B}$ is directly proportional to the storage modulus and can therefore serve as a measure of a material’s elasticity, where a greater modulus (generally stiffer material) corresponds to a higher BFS. The longitudinal modulus should not be confused with Young’s modulus, which describes unconfined compression, with implications for Brillouin microscopy reported previously.^22^ In anisotropic materials the sound wave velocity and hence the longitudinal modulus are dependent on the direction of the wave propagation. Therefore, Brillouin microscopy can probe direction-dependent elasticity via choosing appropriate scattering geometry and the incident light direction.^23^

On the other hand, the full width at half maximum of the scattered light, so called Brillouin linewidth (BLW, $\Gamma_{B}$), is linked to the loss modulus ($M^{\prime \prime }$) and the phonon wave vector $q=4\pi n/\lambda_{0}$ as follows:

$$\Gamma_{B}=\frac{M^{\prime \prime }q^{2}}{{\rho \Omega }_{B}}.$$ (2)

Here $M^{\prime \prime }$ represents losses incurred by phonons which can be related to either viscous damping or structural heterogeneity of the material. Provided that material density and refractive index can be measured or estimated,^24^ the Brillouin frequency shift $\Omega_{B}$ and linewidth $\Gamma_{B}$ can be used to derive the complex longitudinal modulus of the material:

$$M^{*}=M^{\prime }+iM^{\prime \prime }.$$ (3)

Due to the symmetry of photon-phonon interaction, the Brillouin light scattering process can go either with the generation of a new phonon (and annihilation of a photon) producing a down-shifted Stokes peak ($f=-\Omega_{B}$) or, vice-versa, with annihilation of a phonon leading to an up-shifted anti-Stokes peak ($f=+\Omega_{B}$)^25^ [Fig. 1(b)]. In the light spectrum, these interactions appear as symmetric side bands set around the central laser peak at f=0. All of this is measured all-optically, without exertion of force upon the target medium, in principle enabling mechanical mapping of biological samples *in vivo* and *in situ* without physical contact or labeling.^24^

**Fig. 2:**
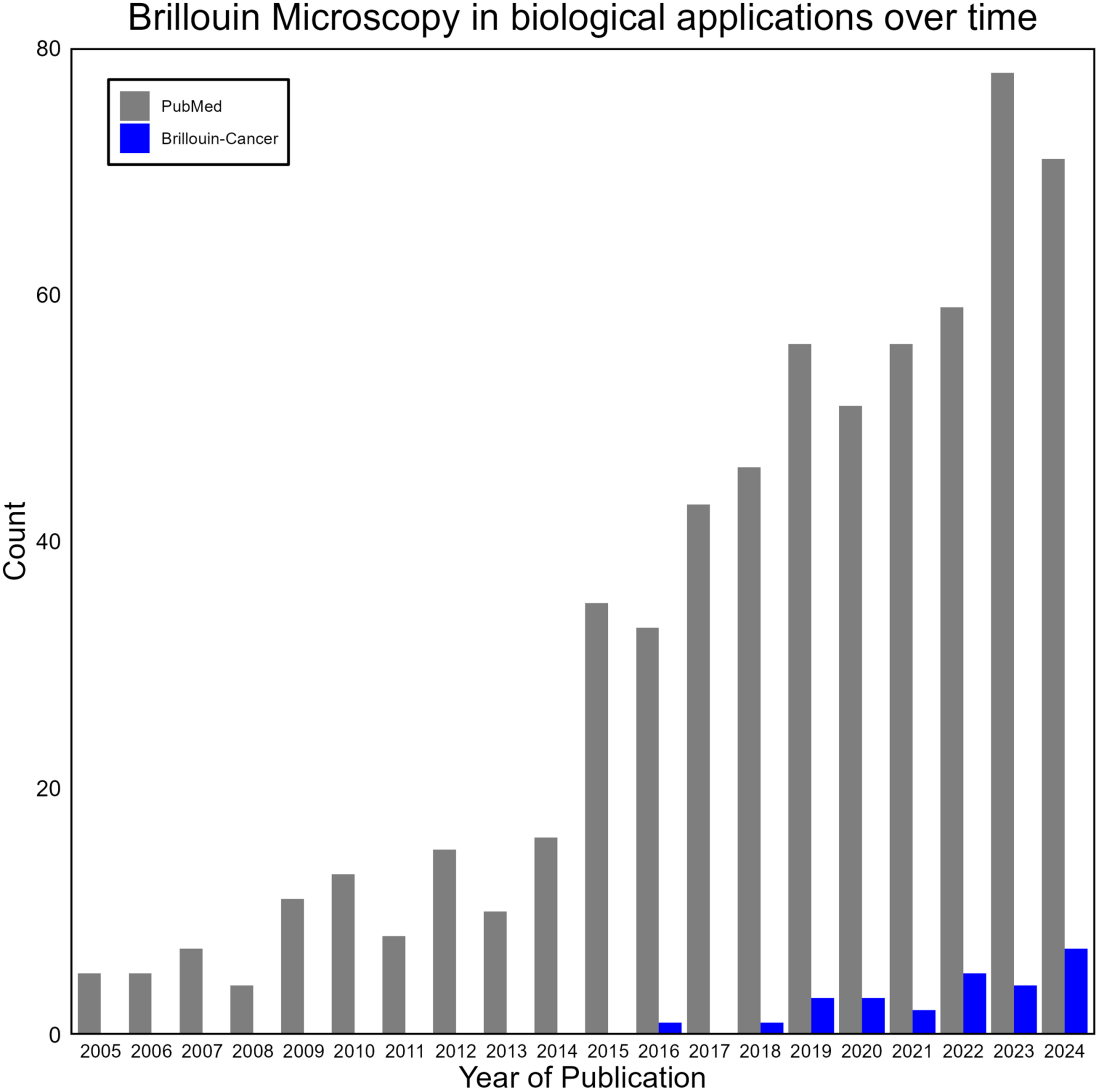
Timeline of Brillouin microscopy development constructed from a query of NIH’s PubMed database (gray) and a local collation of all known published Brillouin microscopy reports applied directly to cancer research (blue). The PubMed query searched for the terms “Brillouin imaging,” “Brillouin microscopy,” and “Brillouin microspectroscopy,” mentioning the technique anywhere within the paper.

Brillouin light scattering spectroscopy is a well-studied technique that has been applied within the material sciences and solid-state physics since the 1930s.^26^ It was not until the mid-2000s, when the method evolved from point measurements to spectroscopic imaging, that the technique was adopted for mapping biologically relevant materials, such as tissues, hydrogels and cells.^27^ The idea of using Brillouin microscopy as a method of differentiating between cancerous and healthy tissues as well as identifying tumor margins was first reported in 2016,^28^ after which its application to cancer research and diagnostics expanded (Fig. 2). By this time, methods of mechanical mapping, such as atomic force microscopy (AFM) indentation,^29^^,^^30^ had already been established. Compared with AFM, Brillouin microscopy is non-contact, label-free, and can conduct three-dimensional (3D) scanning beyond the surface of a sample. The penetration depth of Brillouin microscopy is typically on the order of a few hundred micrometers and limited by light scattering and absorption within the sample, similar to other optical imaging modalities. Due to these properties, Brillouin microscopy’s feasibility has been investigated thoroughly, and the method has shown great promise not only on its own but in combination with other imaging and spectroscopic modalities.^24^^,^^31^^–^^35^ Currently, Brillouin microscopy is an increasingly popular method of mechanical probing, entering the realm of clinical adoption and standardization.^36^

**Fig. 3:**
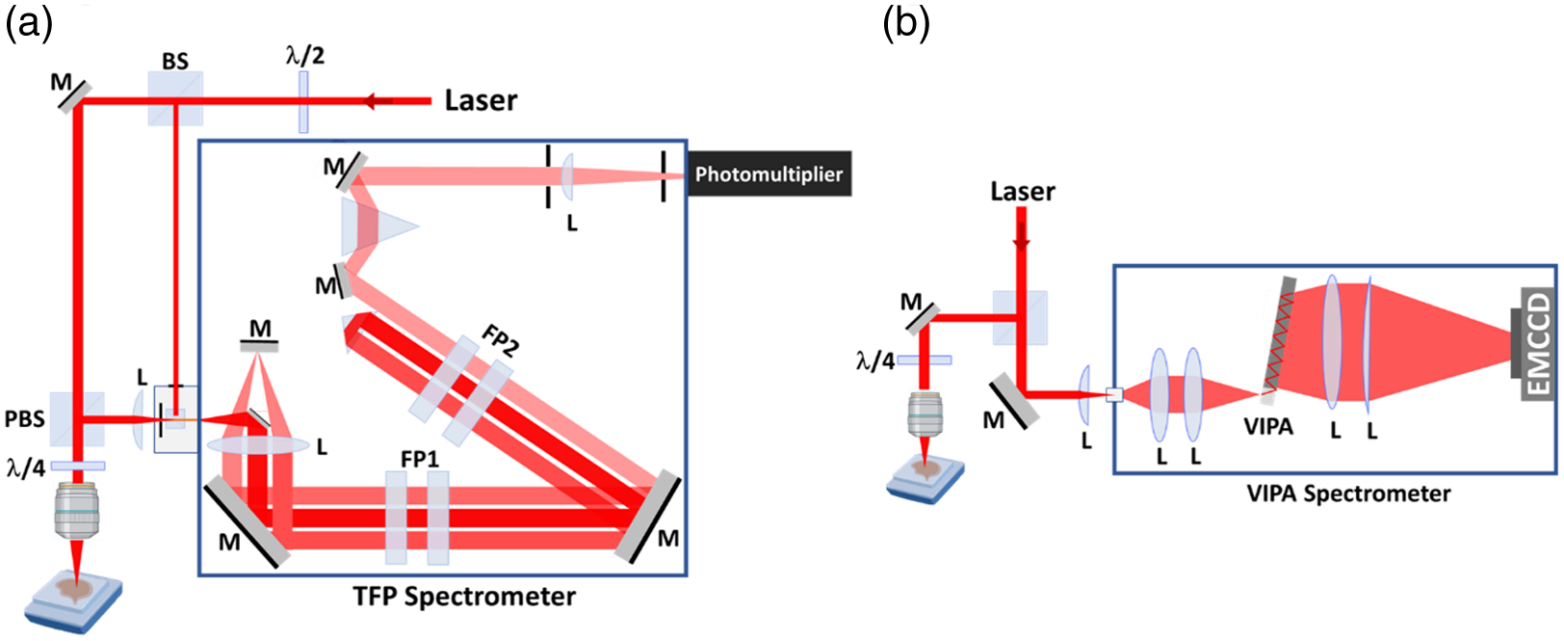
Types of apparatus for Brillouin microscopy: (a) a microscopy setup assisted with a tandem Fabry–Perot (TFP) spectrometer and (b) Brillouin microscopy based on virtually imaged phase array (VIPA) spectrometer. M, Mirror; L, Lens; BS, beam splitter; PBS, polarization beam splitter; FP, Fabry–Perot.

A basic Brillouin microscopy setup consists of three essential components: a narrow-linewidth (<1  MHz), frequency-stabilized laser, a high-resolution spectrometer, and a confocal imaging system (Fig. 3). Due to the low efficiency of spontaneous Brillouin light scattering process^37^ and small BFS in the GHz range, for a long time the traditional approach to Brillouin light scattering instrumentation relied on high-sensitivity and high spectral resolution spectrometers, such as scanning tandem Fabry–Perot interferometers (TFPIs).^38^ More recently, Scarponi et al.^39^ designed a combined Brillouin-Raman spectrometer specifically for measurements in semi-opaque samples, capable of achieving a signal-to-noise (SNR) contrast of 150 dB. Despite superior spectroscopic performance and reconfigurability, TFPIs have limited acquisition speed of 0.5 s per spectrum as they rely on piezo-driven mirror movement. As a result, imaging applications requiring the collection of over 1000 point-spectra across a sample can take up to several hours to complete. To overcome this issue, a non-scanning version of a Brillouin spectrometer was developed by Koski and Yarger^40^ where angular dispersion of light in a tilted Fabry–Perot etalon produced the desired interferogram. This concept was further developed into Virtual Imaged Phased Array (VIPA) reducing instrument complexity and cost, although at the expense of compromised sensitivity (SNR∼30  dB) and reduced spectral resolution in comparison with TFPIs. Since then, various VIPA-based spectrometer designs have been proposed to improve signal extinction and imaging speed, with state-of-the-art instruments reaching beyond 60 dB sensitivity and approximately 20 ms acquisition time per single spectrum.

Another critical factor is the choice of the laser wavelength – a shorter wavelength improves scattering efficiency ($\propto \lambda^{-4}$), whereas 660 to 780 nm is typically favored in biological applications to minimize photodamage and autofluorescence. Brillouin microscopes often operate in a confocal epi-illumination mode, providing submicron spatial resolution (<0.5  μm lateral and <1.5  μm axial) when using high numerical aperture objectives (NA > 0.6). However, spatial resolution may also be limited by the acoustic phonon mean free path within the sample.^41^ For further details on Brillouin microscopy instrumentation, including stimulated Brillouin scattering approaches, we direct the reader toward recently published reviews.^22^^,^^42^

## 3 Analysis of Published Results Categorized by Sample Type

**Fig. 4:**
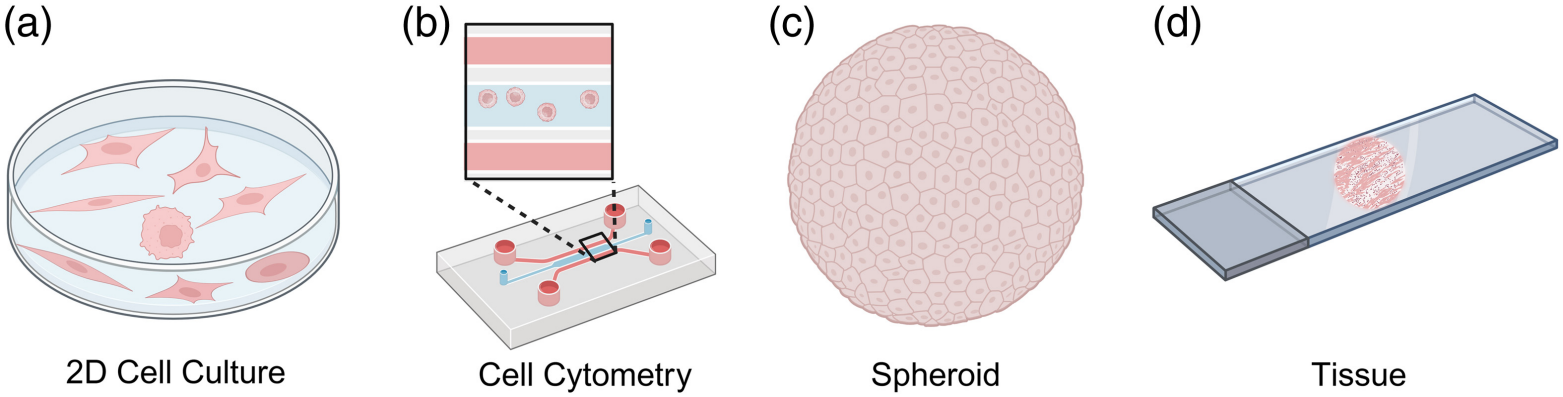
Overview of various cancerous sample models analyzed using Brillouin imaging: (a) 2D cell cultures, (b) cell flow cytometry using microfluidics, (c) spheroids, and (d) animal or human tissues. All diagrams in Fig. 4 were made in Biorender.

Changes in the biophysical properties of cancer cells and their microenvironments, such as viscoelasticity, play a key role in cancer progression.^43^ In this section we will review the published reports which detail the application of Brillouin microscopy to cancer research by categorizing this data by the type of biological model. As cellular functions are closely linked with cell morphology and the extracellular environment, comparative analysis can only be performed on similar types of samples. Thus, across all the published data we identified the following sample types: (1) single cells in two-dimensional (2D) cultures, (2) single cells in microfluidics, (3) cellular spheroids and (4) animal/human tissues (see Fig. 4).

To make a meaningful comparison between individual reports within each category, we present the data for BFS and BLW in terms of percentile changes from the average or the respective control group. We note that changes in BFS and BLW should not be equated to changes in sample stiffness (Young’s modulus), thus a few percent difference in BFS may correspond to a significant (>10%) stiffness change (to be confirmed by complimentary techniques). For example, in a comparative study using Brillouin microscopy and Atomic Force Microscopy, 7% increase in BFS was found to represent 3-fold growth in the Young’s modulus.^44^ On average, the precision of Brillouin microscopy measurements is typically reported to be within a fraction of a percent (<10  MHz for BFS of ∼6  GHz), making changes in BFS and BLW of a few percent significant. Notably, Scarcelli et al.^44^ reported that a 0.05% change in the longitudinal modulus-equivalent to a ∼5  MHz shift in Brillouin frequency-corresponded to an approximate 2% change in Young’s modulus, illustrating the high sensitivity of Brillouin microscopy to subtle biomechanical variations.

In addition, it should be noted that the absolute values of BFS and BLW scale with experimental parameters, e.g., the wavelength of the laser and instrument calibration. In rare cases where only the absolute value of the frequency shift is noted, efforts are made to convert the data to the same wavelength of the probing laser.

### 3.1 2D Cell Cultures

Across all Brillouin microscopy studies applied to mapping mechanical properties of 2D cell cultures, consistent findings demonstrate that changes in cell stiffness are closely linked to cancer progression. Namely, cancer cells grown on stiffer surfaces showed higher Brillouin shifts (+7.1%), reflecting increased intracellular stiffness and reduced deformability.^12^ Phenotypic transitions, such as epithelial-to-mesenchymal transition (EMT), were associated with increased cellular stiffness, as indicated by a +0.47% higher Brillouin shift in CTGF-knockout (CTGF-KO) cells compared with wild-type (WT) controls. CTGF-knockout was performed by CRISPR technology and led to reprogramming of cytoskeletal remodeling during EMT. Overall, CTGF-KO cells were found to have higher actin-staining than WT cells, correlating well with increased BFS. This increase was partially reversed (−0.16%) following the experimental addition of recombinant CTGF.^45^

Subcellular biomechanical mapping revealed a stiffness gradient within individual cells, with the nucleus exhibiting a Brillouin shift +4.3% higher than the surrounding regions, followed by the perinuclear region (+3.73%) and cytoplasm (+2.35%).^46^ When the cytoskeleton was disrupted, using 1  μM cytochalasin D (a pharmacological inhibitor of actin), the Brillouin shift decreased by −0.56%, indicating that cells became softer and more flexible. These findings could be helpful in understanding cellular processes such as transendothelial migration, where both whole cells and their nuclei become softer (reporting BFS decreases by −0.64% and −0.80% respectively), facilitating movement through barriers.^47^

Comparisons between non-tumorigenic and metastatic cancer cell lines confirmed that metastatic cells consistently exhibit lower Brillouin shifts. For example, MCF10A, a non-tumorigenic human breast epithelial cell line, demonstrated nuclear and cytoplasmic shifts of +0.57% and +0.58% higher, respectively, than those in MDA-MB-231 metastatic cells.^43^

Overall, these studies suggest that Brillouin microscopy can be valuable for detecting micromechanical changes in cancer cells, particularly in 2D cultures, with softer cells often linked to more aggressive and invasive cancer phenotypes.

### 3.2 Cell Flow Cytometry

Brillouin microscopy in combination with microfluidic channels and flow cytometry have been used to analyze cancer cells in fluidic environments to investigate cellular biomechanics. Microfluidic models are valuable as they mimic complex key aspects of the tumor microenvironment,^48^ thereby enabling detailed studies of cell behavior under physiologically relevant conditions.

Wisniewski et al. reported that invading cancer cells modulate their migratory behaviors in response to microchannels with different confining geometries – a phenomenon called dorsoventral polarity.^49^ Using Brillouin microscopy, they observed that the nuclear BFS of cells in vertical confinement is +0.8% higher than cells in lateral confinement. It was further demonstrated that this confinement-induced nuclear stiffening plays a crucial role in regulating cancer cell migration.

Rosvold et al.^50^ demonstrated that normal breast epithelial cells (MCF10A) exhibit higher BFS in both the nucleus (+2.57%) and cytoplasm (+2.42%) compared with the metastatic MDA-MB-231 breast cancer cells, indicating greater stiffness in normal breast cells. This contrast can be attributed to cytoskeletal alterations, reduction in adhesion and enhanced invasiveness of malignant cells. In addition, in both cell lines, the nucleus displayed higher BFS than the cytoplasm, which is in good agreement with other 2D and 3D cellular studies. A complementary study investigating epithelial ovarian cancer cells (NIH: OVCAR5) demonstrated that environmental conditions modulate tumor cell stiffness. Tumoroids cultured under continuous flow conditions exhibited reduced BFS values of −0.79% compared with those maintained under static conditions. In addition, the influence of media change in nodules was assessed over a seven-day period. Daily media changes throughout the study resulted in a −0.94% decrease in BFS compared with nodules with no media change. Notably, a single media change on day 7 did not produce a statistically significant difference in BFS compared with daily media changes.^48^ This indicates that alterations in nutrient levels have a negligible impact on the mechanical properties of tumor organoids *in vitro*.

### 3.3 Spheroids

Spheroids are increasingly used in cancer research, as they provide a more physiologically representative model of 3D *in vivo* tumor and tissue environments compared with conventional 2D monolayer cell cultures. Unlike 2D models, spheroids can emulate complex cell-cell interactions and demonstrate a more accurate replication of the structural and biochemical characteristics of the TME.^51^^,^^52^ This enhanced physiological relevance enables a more accurate assessment of cellular behavior and biomechanical properties, including tumor progression, heterogeneity, and response to therapeutics. However, directly quantifying the mechanical properties of cells within a spheroid remains a significant challenge for conventional technologies, which either probe only the superficial layer by physical contact^53^ or require an invasive procedure such as microbead injection.^54^ In this regard, Brillouin microscopy offers a promising solution to this technical limitation due to its non-contact and non-invasive nature.

#### 3.3.1 Effects of chemotherapeutic treatments on tumor spheroids

Brillouin microscopy has been used to assess the biomechanical response of spheroids to chemotherapy drugs and to evaluate real-time drug-induced phenotypic changes, such as stiffness. Recent studies have demonstrated chemotherapy treatment induces a progressive softening in tumor spheroids over the course of the experiment.^55^^–^^57^

A study on colorectal cancer spheroids (HCT116) treated with 5-fluorouracil (5-FU), a drug that targets the S-phase of the cell cycle by inhibiting DNA synthesis and disrupting RNA function,^58^ revealed a time-dependent decrease in BFS over two days across all regions of the spheroid, indicating mechanical softening in response to chemotherapy.^55^ By day 2, the outer region (rim) of the spheroids showed the greatest decrease in BFS of −3.23% compared with the intermediate and center regions which had decreases of −1.22% and −1.06%, respectively. These findings suggest a regional effect where a gradient of drug penetration is observed, with the outer regions of the spheroid showing greater susceptibility to chemotherapy-induced mechanical changes than the core.

Temozolomide (TMZ), a chemotherapeutic agent used in the treatment of glioblastoma, also induced mechanical softening in spheroids over a seven-day period. LN229 spheroids treated with TMZ exhibited a significant reduction in BFS (−1.61%) by day 7.^56^ Spheroids composed of only human astrocytes (HA) followed a similar trend, with a slight decrease in BFS (0.82%). Notably, co-cultured LN229 and HA spheroids also demonstrated reduced BFS values of −1.12% by day 7, which was less than the change observed for monocultured LN229 spheroids. This suggests induced resistance to chemotherapeutic treatment via TMZ. In addition, a study by Guerriero et al.^57^ investigated the use of nanoparticles (NPs) as therapeutic agents in multicellular tumor spheroids (MCTS), with a focus on the spheroid core. Over a four-day treatment period, NPs induced a gradual decrease in BFS, indicating the progressive loss of mechanical integrity in the MCTS. Although no significant changes were observed for the first 48 h, a steady decline followed, reaching a −1.86% reduction by day 4.^57^ Altogether, these studies reveal the consistent biomechanical softening of tumor spheroids in response to various chemotherapeutics.

#### 3.3.2 Biomechanical evolution of tumor spheroids during growth

**Fig. 5:**
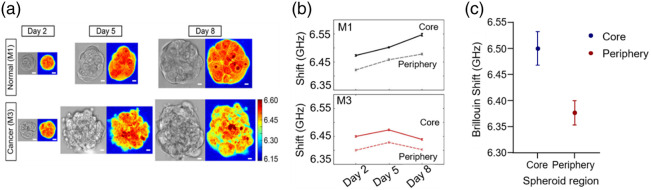
Biomechanical properties of cancer spheroids measured by Brillouin microscopy at 660 nm. (a) Brillouin and bright-field images of a normal spheroid (M1) and a cancerous spheroid (M3) acquired on days 2, 5, and 8. Scale bar: 10  μm. Image reproduced from Hilai et al.^60^ (b) Average Brillouin frequency shifts of the core and periphery regions of M1 (top) and M3 (bottom) spheroids. Image reproduced from Hilai et al.^60^ (c) Brillouin frequency shift (GHz) at the core and periphery regions within spheroids samples (based on separate studies by Cheberkanov et al.,^61^ Hilai et al.^60^ and Margueritat et al.^55^).

Brillouin microscopy has been employed to track the biomechanical evolution of spheroids over time, revealing mechanical changes in tumor spheroids compared with their healthy counterparts. In one study,^59^ the average BFS was quantified for M2 tumor-like breast tissue spheroids (formed from a tumor cell line MCF10AT1k.cl2) and M1 healthy tissue spheroids (formed from the breast epithelial cell line MCF10A) at early (i.e., day 2) and later (i.e., day 5) stages of culture. It was observed that, although the average BFS of both kinds of spheroids increased by day 5, tumor spheroids exhibited a greater increase (+2.01% for M2 vs +1.66% for M1), suggesting more pronounced biomechanical changes associated with tumor growth. Similarly, another study^34^ investigated the mechanical state of metastatic breast cancer cells (MCF10CA1h) over a five-day period, where a steady increase of +1.18% in BFS was reported. To minimize potential artifacts arising from the use of different cell populations in longitudinal studies, a recent investigation tracked the mechanical changes of the same cells over the course of eight days [see Fig. 5(a)].^60^ Consistent with previous reports, an increase in BFS was observed in both healthy (MCF10A) and metastatic cancer cells (M3: MCF10CA1h) up to day 5. Intriguingly, although M1 healthy cells continued to show an increase in BFS by day 8, M3 cancer cells exhibited a significant decrease, resulting in an average BFS comparable to that measured on day 2 [Fig. 5(b)]. This mechanical aberration of tumor spheroids on Day 8 is thought to be related to the reduced intercellular adhesion that could facilitate cancer metastasis.^62^

#### 3.3.3 Spheroid heterogeneity

Several studies have demonstrated spatial variations in mechanical properties within spheroids, where the “core region” of spheroids exhibits a higher BFS when compared with the “periphery region”^43^ [Fig. 5(c)]. In 4T1 murine fibroblast cell spheroids, a statistically significant increase in stiffness was observed in the core region compared with the outer periphery, with a difference of +2.80%.^61^ Similarly, Hilai et al.^60^ segmented spheroids into two regions to analyze the spatial distribution of intratumoral elasticity. Both cancerous (MCF10CA1h) and non-cancerous (MCF10A) spheroids consistently exhibited higher BFS in core regions relative to the periphery throughout an 8-day period. By day 8, the BFS of the core was higher than that of the periphery by +0.62% in cancer and +1.23% in healthy spheroids [Fig. 5(b)], which suggests that the mechanical heterogeneity of the spheroids originates from the biomechanical organizations at their periphery rather than at their core.^60^ Although Margueritat et al. reported the effects of 5-FU on HCT116 cells, their findings also suggested insight into regional mechanical differences, where the central region (core) exhibited greater BFS than the peripheral (rim) region.^55^ Collectively, these studies support the presence of a mechanical gradient from the spheroid’s core to its periphery, which is consistent with the results acquired by optical tweezer approaches.^54^^,^^55^^,^^60^^,^^61^ We note that the distribution of mechanical properties across the spheroid volume would depend on the spheroid size, with larger spheroids possibly developing a necrotic core which may affect the mechanical reading. Alternatively, cell density typically increases dramatically from periphery to core of spheroids due to continued cell division within confined spaces, which would affect observed mechanical readouts. Another effect that may influence the reading of BFS and linewidth at the interface between the spheroid and the surrounding medium is linked to phonon reflections at the boundary and averaging of phonon properties across the interface between media and the spheroid.^63^

### 3.4 Tissues

Although 2D and 3D lab-grown cell cultures have become popular modes of sample preparation in cancer research, Brillouin microscopy’s feasibility ultimately needs to be tested on animal and human tissues to validate its realistic efficacy in medical imaging and diagnostics. The analysis of tissue, both excised and *in vivo*, provides an accurate picture of a tumor’s growth and interaction with its surrounding environment, complementing the thorough exploration made regarding cancer cell behavior individually and in clusters. For Brillouin microscopy to be considered a viable diagnosis method, protocols for differentiating tumor tissue from its healthy surroundings must also be established.

Brillouin microspectroscopy was applied to differentiate malignant melanoma from its surrounding healthy tissue, as well as from non-malignant regressive tumors.^28^^,^^64^ The biological model used in these studies (Sinclair miniature swine) is unique as the animal develops melanoma tumors at birth, which then regress in the weeks after. It was found that the regular tumor had BFS approximately +7.28% higher than the surrounding healthy tissue, and the regressing tumor showed more modest BFS changes of approximately +1.76%. This finding agrees with other published reports suggesting that melanoma is stiffer than healthy tissue.^65^^,^^66^

**Fig. 6:**
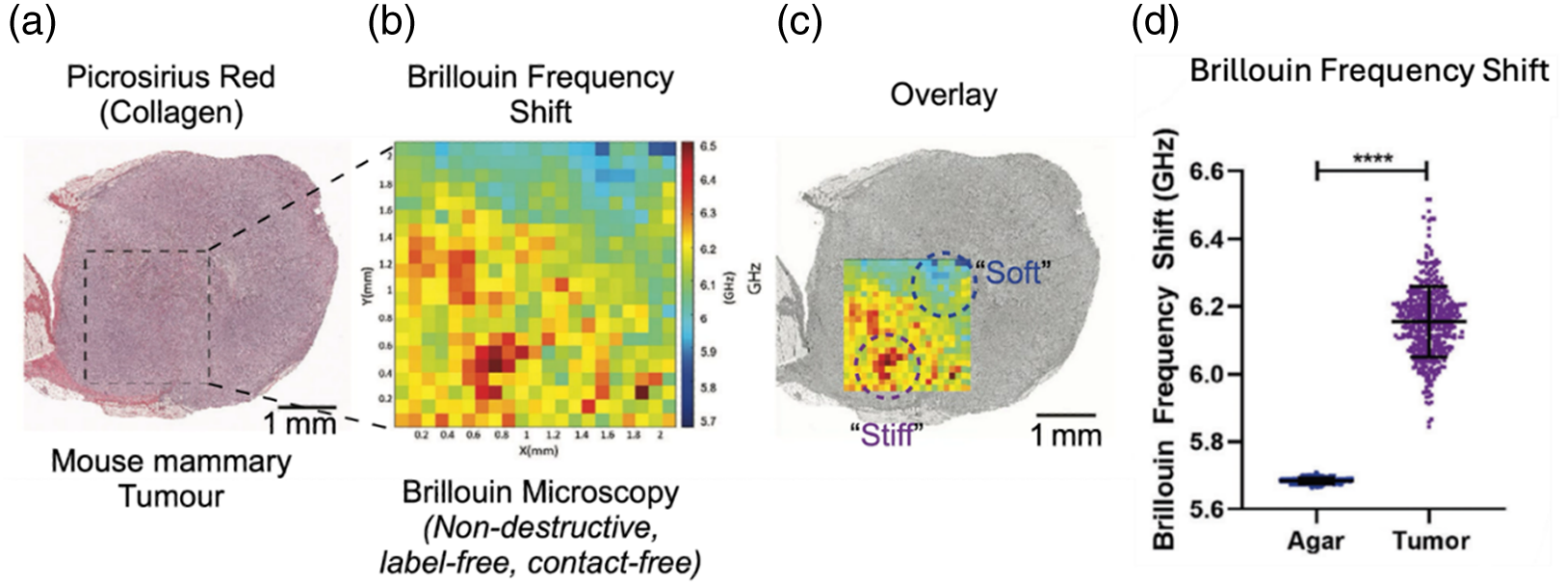
Graphics adapted from the study by Filipe et al.^12^ (a) Histological (H&E) staining of mouse murine mammary tumor brought under a Brillouin Microscope equipped with 660 nm laser source (scale bar = 1 mm). (b) Zoomed in 2D Brillouin frequency shift heatmap corresponding to the same surface region of the tumor shown in image (a). (c) Overlay of images (a) and (b) highlighting “soft” and “stiff” regions as identified by the 2D Brillouin frequency shift heatmap. (d) Brillouin frequency shift within a $2\;{\mathrm{mm}}^{2}$ section of the tumor, compared with the agar embedding material (**** = p<0.0001).

Recently, a study investigating the biomechanical heterogeneity of aggressive triple-negative breast cancer (Fig. 6) found a variation of 7.56% in BFS across a murine mammary tumor.^12^ Physical tumor heterogeneity is presently an active topic of research^5^^,^^9^^,^^67^ with far-reaching implications for tumor pathophysiological development and treatment efficacy. As Brillouin microscopy exhibits sub-cellular resolution, the technique can identify variability in viscoelasticity at micrometer scales in three dimensions, which has subsequent effects on biological heterogeneity of cell behavior and phenotype (both malignant cells and non-malignant cells). A scan of a $2\;{\mathrm{mm}}^{2}$ section of a tumor with 100 micrometer steps yielded a distribution of BFS values ranging from 5.7 to 6.5 GHz across the tissue [Fig. 6(d)]. This significant variance is 10 times greater than that seen in the supporting material, agar, a mechanically homogenous material in which the tumor was embedded. Spatially distinct “soft” and “stiff” regions identified by this distribution are highlighted in Figs. 6(b) and 6(c). In comparison, standard histopathology staining [Fig. 6(a)] did not reveal any difference between the tissue regions suggesting that Brillouin microscopy may have a sensitivity advantage in detection of early tumors.^68^

## 4 Discussion and Perspectives

### 4.1 Advantages and Limitations of Brillouin Microscopy for Cancer Research

We start by discussing the advantages and limitations of the Brillouin microscopy technique in application to cancer research and diagnostics. One invaluable advantage of Brillouin microscopy is its non-contact and label-free nature which makes it possible for the technology translation to the medical field of diagnostics and therapeutics. Currently, clinical studies on the application of Brillouin microscopy in ophthalmology are underway in the USA, with methods being tested on human patients.^69^ Brillouin microscopy offers a three-dimensional resolution of ocular tissues and reveals spatial variation in their mechanical properties by measuring the longitudinal modulus, making it a promising tool for corneal diagnostics and treatment monitoring, particularly for conditions such as keratoconus.^70^ Although not directly related to cancer, this demonstrates the translational potential of Brillouin microscopy in medical diagnostics. Accessing measurements within the human body requires the development of endoscopic approaches to Brillouin imaging, which are also underway with some preliminary designs and proof-of-principle demonstrations reported within the last decade.^71^^–^^73^ With the improvements in Brillouin fiber probe collection efficiency and device packaging, we foresee *in situ* applications of Brillouin endoscopy to diagnostics and screening of many diseases, including cancer, without the need for tissue biopsy. AI-driven approaches to image recognition and data analysis will likely speed up this technological translation in the same way this is taking place in MRI and CT imaging today, potentially allowing precisely establishing cancer margins in real time during the surgery.

Many of the reports published to date and reviewed in this article note the small changes in Brillouin frequency shift as the result of a drug therapy application, cellular growth cycle or morphological transformation. This, so-called narrow dynamic range, presents a considerable challenge to the users and adopters of this technology, setting stringent requirements for the stability and repeatability of the measurements. In addition, it does not help that mechanical properties measured with Brillouin microscopy are not necessarily specific to material components, but rather an average measure of several components at once due to the technique’s limitation on spatial and spectral resolution. A high percentage of the Brillouin microscopy systems used for imaging tumor samples to date utilize VIPA-based spectrometers. These, unfortunately, have limited spectral resolution, typically 0.5 to 1 GHz which complicates absolute quantification of BLW^32^^,^^42^—the parameter essential for assessing viscose component of the material’s viscoelastic response. Although tandem Fabry–Perot interferometers (TFPs) provide improved resolution, their acquisition speed is too slow for many biological applications, including imaging of dynamic, live biological systems.^42^ Stimulated Brillouin scattering (SBS) microscopy has recently emerged as a promising alternative, offering both higher spectral resolution and faster acquisition rates, enabling more accurate BLW quantification for complex biological systems.^74^

Unlike Raman scattering, where each molecular bond is assigned a specific frequency, two hydrogel fabrications with significantly different molecular compositions may exhibit the same average Brillouin frequency shift albeit for a different set of physical parameters such as temperature, hydration, and crosslink concentration. Although this may not present a problem from a clinical point of view, Brillouin data analysis and deconvolution of spectral features is significantly more challenging in heterogeneous samples such as tissues, impacting the development of the field and potentially hindering the translation of this technique to biomedical applications. Additional strategies, e.g., moving toward multimodal imaging by combining Brillouin imaging with Raman,^30^ fluorescence, two-photon or other imaging modalities that can improve the specificity of findings, will be advantageous and increase the diagnostic potential of Brillouin microscopy overall.

### 4.2 Brillouin Data Analysis, Post- and Pre-processing Using Machine Learning, AI, and Statistical Methods

Since the introduction of Brillouin imaging in 2005,^40^ continuous hardware improvements have greatly enhanced the sensitivity and resolution of Brillouin spectra. Current high-end systems achieve acquisition times on the order of 0.05 to 1 second per spectrum,^75^ a marked advancement from earlier implementations. However, this rate remains insufficient for *in vivo* applications, where thousands of spectra may need to be recorded within the same time frame to form a single image. The resulting low data acquisition rate poses a critical bottleneck for real-time imaging and hampers the broader adoption of Brillouin microscopy in fast, dynamic biological studies.

A significant challenge lies not only in data collection but also in the computationally intensive processing of the acquired hyperspectral data. Traditionally, Brillouin spectra are analyzed using standard curve-fitting algorithms, such as Lorentzian or Damped Harmonic Oscillator (DHO) functions,^21^ to extract peak positions and linewidths. Although these methods are robust, they can be slow and sensitive to noise, making them less suitable for large datasets where high fidelity and rapid processing are both required. Pre-processing steps such as denoising, data normalization, spectral drift correction, and baseline correction are often applied to improve the quality of Brillouin spectra before analysis.^21^ Cell and tissue movement tracking algorithms may also be needed to compensate for natural changes in the sample position (e.g., due to patient breathing or cell migration) over the acquisition time window.

To overcome these constraints, new approaches that integrate advanced software algorithms show considerable promise. Recently, the application of AI and machine learning techniques to spectroscopic imaging has emerged as a powerful alternative to standard curve fitting. AI-driven models can handle noisier data acquired over shorter time intervals and still accurately reconstruct material property maps, thus addressing the trade-off between speed and data quality. By reducing reliance on time-consuming iterative fitting routines, these methods offer 10 to 100 times faster data processing and have the potential to transform the field of Brillouin imaging by enabling near-real-time imaging. Notably, the effectiveness of such AI-based approaches has already been demonstrated in Raman imaging^76^ and more recently in Brillouin microscopy using multivariate analysis methods such as principal component analysis (PCA) and vertex component analysis (VCA), which offer robust performance even in complex biological samples.^77^ Through combined improvements in hardware optimization and algorithmic innovation, it is foreseeable that Brillouin imaging will soon meet the high-speed, high-throughput demands of *in vivo* studies.

Looking ahead, the integration of deep learning approaches shows promise for cancer diagnostics. AI approaches trained on Brillouin images from various cancer types could potentially identify mechanical signatures specific to malignant transformation, tumor grade, or even treatment susceptibility. These AI-based approaches may ultimately enable real-time interpretation of Brillouin data in clinical settings, where rapid diagnostic decisions are critical.

AI technologies have been extensively deployed in the segmentation and classification of medical images for cancer diagnosis in clinical settings. In this context, the unique mechanical features captured by Brillouin imaging offer a promising new class of biomarkers that could improve the sensitivity and specificity of cancer detection. A recent *in vitro* study has shown that biomechanical information derived from Brillouin imaging provides complementary information to the morphological phenotype of tumor spheroids, and the integration of both using a machine learning model can significantly improve the classification accuracy.^60^ Looking ahead, the combined analysis of Brillouin-derived mechanical phenotypes and existing biomarkers through AI technology holds strong potential for advancing high-accuracy cancer diagnosis. Moreover, the use of explainable AI techniques, such as Gradient SHAP and Saliency-map, enables interpretation of model predictions by highlighting regions of interest that drive classification decisions. This interpretability could yield valuable insights into the biomechanical mechanisms underlying tumor progression and inspire the development of novel therapeutic strategies.

### 4.3 Interpretation of Brillouin Data

It is well documented that the mechanical modulus derived from Brillouin microscopy is different from that measured by other technologies such as AFM and optical/magnetic tweezer, in two key aspects.^22^^,^^44^^,^^68^^,^^78^ First, Brillouin microscopy quantifies the longitudinal modulus, whereas other technologies typically measure Young’s or shear moduli. Second, Brillouin microscopy probes the mechanical modulus at high frequencies (in the range of 1 to 30 GHz), in contrast to the low or quasi-static frequencies used in other methods. Therefore, correlations between Brillouin-derived longitudinal modulus and the moduli obtained from other technologies may not be assumed and should be carefully investigated on a case-by-case basis.^68^ Moreover, Brillouin microscopy alone cannot determine the absolute value of mechanical modulus without prior knowledge of the material’s refractive index and density, and direct quantification of these material parameters further increases the technical complexity. Nevertheless, in clinical applications, BFS and BLW, which are direct readouts of Brillouin microscopy, could be an effective metric for disease diagnosis. This potential has been exemplified recently by the application of Brillouin microscopy in subclinical keratoconus detection.^79^ In cancer research, it was demonstrated that cancer cell spheroids can be successfully distinguished from healthy cell spheroids based solely on BFS images.^60^ In the future, we believe that comprehensive studies are needed to fully explore the possibility of employing BFS and BLW parameters as a diagnostic metric in oncology. Such effort could greatly advance the clinical translation and adoption of Brillouin microscopy.

### 4.4 Mechanical Effects of Biological Sample Preparation

A goal for all biological studies is to conduct a test under conditions as close as possible to a particular natural environment. Live tissues are therefore preferred over fixed or frozen excised samples, as such preparation methods could lead to unwanted viscoelastic changes, altering Brillouin readouts. The increasing use of VIPA spectrometers for Brillouin measurements is partially owed to its ability to be integrated with standard confocal and fluorescence microscopy systems, which may already exist in biology labs. More importantly, faster acquisition time of VIPA-based spectrometers compared with scanning Fabry–Perot systems, enables imaging times suitable for live samples. The excision of tissue samples, however, may create matrix damage and changes to the tissue hydration and mechanics, depending on the tissue slicing method, the sample’s final thickness and the buffers used during preparation and imaging. Sectioning via vibratome slicing, for example, is thought to cause the release of tension within muscle and spinal cord tissue, softening *ex vivo* samples, as found in zebrafish by Schlüßler et al.^80^ Chemical changes in tissue post-mortem, such as rigor mortis, may alter biomechanical measurements if preparations aren’t properly made.^81^^,^^82^ Ultimately, *in vivo* measurements without the need for excision may present the best approach and can produce a true snapshot of tissue biomechanics. Currently, designs to allow for *in situ* Brillouin measurements via hand-held and fiber probes are in the proof-of-concept stage.^71^^,^^73^^,^^83^

### 4.5 Promotion of Methodological Standardization

A core difference between a benchtop experimental modality and a clinically accepted method is its standardization. It is critical to develop commonly accepted measurement protocols, data analysis and reporting methods to translate Brillouin microscopy across fields for adoption in biomedicine. The main challenge of developing such protocols in the field of Brillouin microscopy has stemmed from the diversity of measurement approaches (spontaneous, stimulated and impulsive Brillouin scattering techniques all require different apparatus). In addition, to date majority of the equipment for Brillouin microscopy is developed and automated by researchers themselves in-lab. Although turn-key Brillouin instruments are starting to appear on the market, their price point is relatively high with extra requirements for integration with commercially available microscopes which are assumed to be purchased in addition to a Brillouin spectrometer system. Even then, many also require specialized training and/or knowledge which increases the barrier to adoption.

Recently, a consensus statement has been developed as a unified effort by the entire Brillouin microscopy community to bring this research field to a standardized practice.^36^ The aim of this consensus statement is to improve the comparability of Brillouin spectroscopy and microscopy studies by providing reporting recommendations for the measured parameters and detailing common artifacts. Given that most Brillouin microscopy studies of biological matter are still at the proof-of-concept stage, a consensus statement is particularly timely to assure unified advancement. The role of the International BioBrillouin society needs special acknowledgement in driving the standardization effort, with several society’s focused groups working on the development of instrumentation and data analysis techniques with reports recently published.^22^^,^^42^ We believe that broad adaptation of protocols summarized in the consensus statement together with growing commercialization and cost-optimization efforts in Brillouin light scattering instrumentation will create a fertile ground for the technology’s transition to biological and biomedical disciplines, including cancer diagnostics and therapeutics.

## 5 Conclusion

We presented an overview of Brillouin microscopy technology applied to cancer research and diagnostics. We organized all reported findings based on the sample type to observe common trends for each type while still being able to draw parallels between different sample geometries. We believe this is a necessary requirement as the cell microenvironment has a dominant influence on its behavior and health. Despite the differences between individual experiments, we found common aspects across different cancer sample groups, such as tumor heterogeneity present in spheroids and tissues. This suggests that any experimental approach lacking microscopic and 3D access to a model’s mechanical properties may fail to characterize the local evolution of individual cells within the tumor. Brillouin microscopy, however, is a perfect technique to address this challenge as it allows measurements throughout 3D tissue volumes with submicron resolution. Another important finding is the time-dependent evolution of tumor mechanical properties, where the stiffening or softening may be influenced by the stage of cellular growth, the size of the tumor, the nutrients delivered, and other biophysical aspects. Thus, to fully track the evolution of a tumor over time, the chosen method should be capable of longitudinal studies applied to the same live cell(s) or tissue sample repeatedly without eliciting photodamage, modifying cell behavior, or causing cell toxicity. This important and overall challenging requirement can easily be met by Brillouin microscopy due to its damage- and contact-free nature of the measurement. Although significant work is still needed in improving Brillouin measurement speed, data interpretation, and creating shared Brillouin databases for standard biological materials, the field is rapidly progressing in this direction with the adoption of standard reporting protocols and shared measurement methods. We believe, therefore, that these positive changes are around the corner, leading to a broader spread of technology and its translation into clinical cancer research.

## Data Availability

This literature review was conducted by collating published articles on repositories such as PubMed, ArXiv, and Google Scholar, and all data analyzed in this review have been referenced accordingly. Diagrams 1(a) and 4 in this paper were created using Biorender.com. There are no data created for this review that are not publicly known.
